# Evaluation of clinical status, diagnosis, treatment and radiological findings of pulmonary hydatid cyst: 5-years' experience at tertiary lung center

**DOI:** 10.22088/cjim.13.1.44

**Published:** 2022

**Authors:** Kambiz Sheikhy, Ramin Rouhani, Saviz Pejhan, Alireza Sanei Motlagh, Ali Sheikhy

**Affiliations:** 1 Lung transplant Research Center (LTRC), National Research Institute of Tuberculosis and Lung Disease (NRITLD), Shahid Beheshti University of Medical Sciences, Tehran, Iran; 2 Department of General Surgery, Mazandaran University of Medical Sciences, Sari, Iran; 3 Mazandaran University of Medical Sciences, Sari, Iran; 4 Tehran University of Medical Sciences, Tehran, Iran

**Keywords:** Pulmonary hydatid cyst, Radiological findings, Treatment, Thoracic surgery

## Abstract

**Background:**

Hydatidosis is one of the most critical worldwide parasitic zoonotic diseases. The lung is the second most common site of hydatidosis. This study aimed to evaluate the clinical status, diagnosis, treatment, and radiological findings of pulmonary hydatid cyst in patients referred to tertiary lung center.

**Methods:**

From April 2014 to July 2019, patients referred to Masih Daneshvari University Medical Center with the impression of alveolar hydatidosis included. Demographic data of 304 patients were collected including clinical symptoms, laboratory studies, radiological findings, location of the lung involvement, and cyst characteristics. Also, surgical procedures, medical treatments, and post-operative complications were recorded.

**Results:**

Pulmonary hydatidosis was confirmed for 234 patients. 55% of patients were males with the mean age of 45.1±16.6 years. The most common symptoms were cough (59.8%), dyspnea (31.1%), and hemoptysis (26%). Left lung, right lung, and bilateral involvement were reported in 40.1%, 55.1%, and 4.8% of cases, respectively. Cyst perforation (39.8%) was the most common intra-operative finding. Surgical interventions included thoracotomy, rigid bronchoscopy, cyst aspiration, and enucleation. The liver was the most concomitant organ involved due to pulmonary hydatidosis (16.6%). The most common postoperative complication was atelectasis, with the rate of 35.7%. 52.2% of patients were discharged within 10 days after surgery. No mortality was reported.

**Conclusion:**

Sometimes atypical findings in different imaging modalities make the hydatid cyst diagnosis challenging. Although lobe involvement more than 50% has the indication for lobectomy, we conserved lobes with about 70% involvement in our institution, and patients had no postoperative complications.

Hydatid disease or cystic echinococcosis is one of the most important and oldest common diseases between humans and animals, which is caused by a type of parasite from a group of worms called Echinococcus granulosus. Hydatidosis is an economic and health problem for countries where the disease is endemic. Due to the long incubation period of the disease, it can be expected that clinical symptoms will appear in middle ages. The highest prevalence is in the third and fourth decades, because this age range is one of the active periods of human life. However, the infection has similar proportions in almost all age groups (1, 2). The worldwide incidence of hydatidosis is about 1-200 per 100000. It is expected to be more prevalent in rural areas of developing countries where the main occupation is animal husbandry (3, 4). The hydatidosis annual incidence rate in Iran is about 0.61 per 100000 (5). 

The clinical signs of hydatid cyst vary depending on the involvement of different organs. The liver is the most common site for hydatid cysts (65%), followed by the lungs with the frequency of 25%. Also, it can be seen in all parts of the body, including the spleen, heart, or brain. There is a correlation between the patient's age and the site of infection, which means that lung involvement is more common at younger ages, and liver involvement is more common in older people. Some studies found that hydatid cysts in the lungs, brain, and eyes were more common at younger ages (6, 7). Occasionally, a cyst rupture can cause acute symptoms such as severe dyspnea, anaphylactic shock, cyanosis, and cough. Pneumothorax, hydro-pneumothorax, or empyema may manifest when the cyst ruptured into the pleural space. A cyst without acute rupture may cause frequent itching, symptoms of hypersensitivity reaction, and bronchospasm (8). In the present study, we aimed to evaluate the clinical status, diagnosis, treatment, and radiologic findings of pulmonary hydatid cysts in patients referred to Masih Daneshvari Hospital as a tertiary center for lung disease. 

## Methods

This study was designed as a single institution, retrospective descriptional study, and approved by the institutional review board of Shahid Beheshti University of Medical Sciences. From April 2014 to July 2019, patients referred to Masih Daneshvari University Medical Center with the impression of alveolar hydatidosis included. Smoker patients and those with incomplete medical records were excluded. The diagnosis of alveolar hydatidosis was based on clinical manifestations, radiological and laboratory findings. Data including age, gender, primary symptoms, clinical and radiological findings, laboratory results, surgical methods, and post-operative outcomes were collected from hospital records. The diagnosis of pulmonary hydatidosis was based on clinical signs and symptoms, history of exposure to sheep or dogs, and radiological findings. According to radiological findings, anatomical features of the cyst and involved organs were measured. Surgical treatments, including rigid bronchoscopy, cyst aspiration, thoracotomy, and enucleation of the cyst, were considered when the patients had no contraindication for surgery.

**Study registration and ethics: **Our study was conducted according to the Helsinki Declaration. The study protocol was approved by the Research Ethics Committee of Shahid Beheshti University of Medical Sciences, (IR.SBMU.NRITLD.REC.1399.047). All participants gave written informed consent before surgery.

**Statistical analysis: **The numerical variables were expressed as mean and standard deviation (SD). For quantitative variables, depending on the distribution, results were expressed as mean ± standard deviations (SD) or median values and inter-quartile ranges (IQR). A p-value less than 0.05 was considered being statistically significant. All the statistical analyses were performed using the SPSS Version 24.0 program (IBM corp., Chicago IL, USA).

## Results

**Figure 1:**
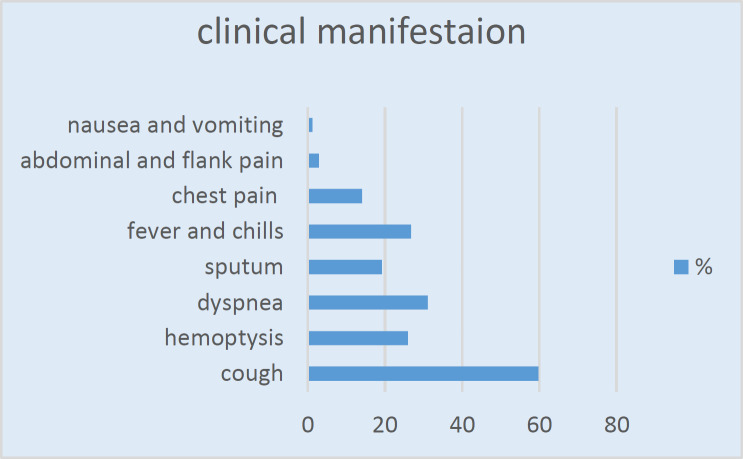
Clinical presentation of patients with pulmonary hydatidosis

From 304 consecutive patients who were highly suspicious of hydatid cyst, 234 patients were confirmed for pulmonary hydatidosis. 130 (55.6%) patients were males, and the mean age was 45.19±16.62 years (ranging from 11 to 81 years). cough, dyspnea, and hemoptysis were the most common clinical manifestations with the rates of 59.8%, 31.1% and 26%, respectively. Chest pain was reported in 33 patients (14.1%), followed by abdominal and flank pain in 7 patients (2.9%). Also, 63 cases (26.8%) had fever and chills, and 45 patients (19.2%) had a history of sputum production (figure 1). 

**Figure 2:**
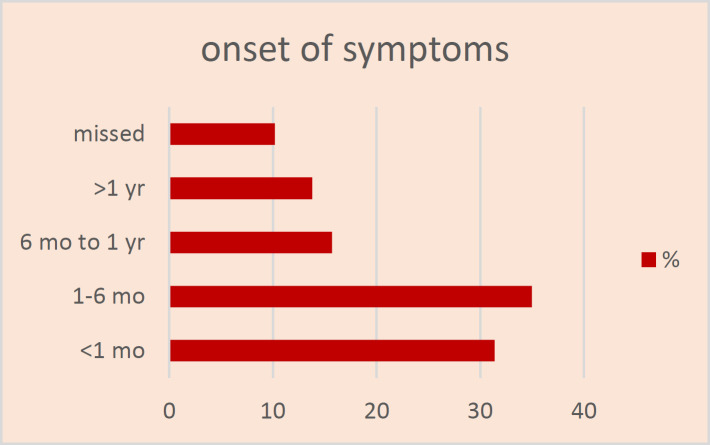
Time interval between primary exposures to onset of clinical symptoms

In some cases, patients had multiple clinical signs. Other signs, including chocking sensation, weight loss, fatigue, dizziness, and diaphoresis, were also reported due to pulmonary hydatidosis. The time interval between primary exposure to onset of clinical symptoms was less than one month for 66 patients (31.4%), one to six months for 82 patients (35%), six months to 1 year for 33 patients (15.7%), and more than one year for 29 patients (13.8%). It was noted that clinical symptoms were underdiagnosed in 24 patients (10.2%) (Figure 2). 

**Figure 3:**
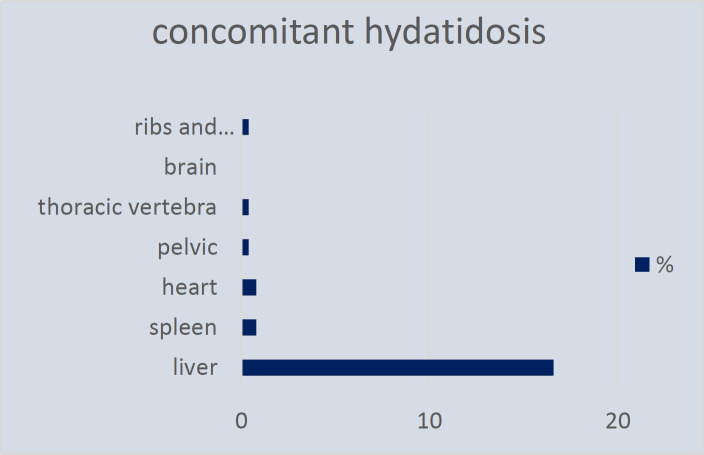
Concomitant organ hydatidosis in patients with pulmonary hydatid cyst

Based on radiologic findings, the size of the cysts ranged from 11 to 150 millimeters. According to pre-operative radiologic data, cysts larger than 120 mm, or cysts with multilobar involvement were considered as huge cysts. According to radiological findings, cysts were classified as simple cyst without internal architecture (14.5%), cyst with daughter cysts (34.6%), calcified cyst (2.5%), and complicated or ruptured cyst (41.8%). As shown in figure 3, the most concomitant involved organ was liver, including 39 cases (16.6%). Subsequently, 2 cases of spleen (0.8%), 2 cases of cardiac (0.8%), 1 case of pelvic (0.4%), 1 case of thoracic vertebra (0.4%), and 1 case of ribs and paraspinal muscle (0.4%) hydatidosis was detected. There was no case of brain involvement. According to laboratory results, 48 (20.5%) patients had anemia, and 121 (51%) patients had impaired creatinine levels (normal ranges considered as 0.7–1.4 mg/dL). Aspartate transaminase (AST) and alanine aminotransferase (ALT) levels were higher than normal (>35 IU/L) in 24 (10.2%) and 31 (13.2%) cases, respectively. 

**Figure 4:**
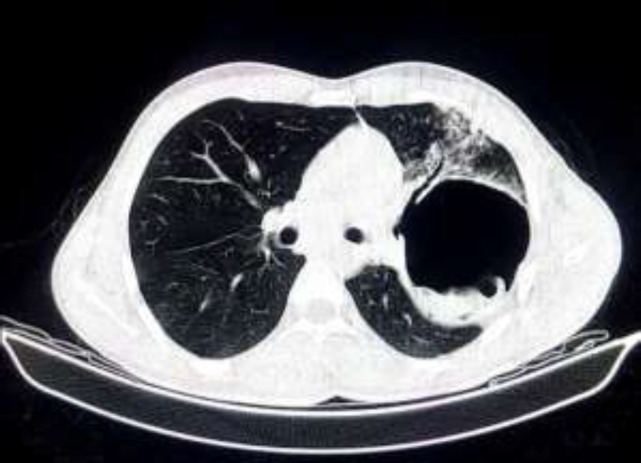
Computed tomography of the chest in 36-year-old male patient presented with history of severe coughing, dyspnea, and productive salty sputum. Note the ruptured cysts into the left lobar bronchus

The anatomical and clinical features of cysts and involved lungs are noted in table 1. From a total of 234 pulmonary hydatidosis, there were 6 (2.5%) cases of recurrent pulmonary hydatidosis. The left lung was involved in 94 (40.1%) cases, the right lung was involved in 129 (55.1%) cases, and bilateral involvement was reported in 11 (4.8%) cases. Altogether, in 28 (11.9%) cases, the cyst was intact. Perforation (39.8%), huge cyst (8.9%), infected cyst (2.9%), and multiple cysts (2.1%) were the most common findings. Rupture of the pulmonary hydatid cyst can occur into the pleural cavity or bronchus. Rupture of the cyst into bronchus can lead to severe cough, dyspnea, and chest pain. Some patients reported a history of productive salty sputum (figure 4). 

**Table 1:** The anatomical and clinical features of cysts and involved lungs

total	N/A	recurrent	huge	multiple	infected	perforated	intact	CYST LUNG	
28 (11.9%)	9	0	4	1	0	8	6	LUL	Left
47 (20.1%)	17	0	4	0	1	23	2	LLL	
19 (8.1%)	8	1	2	1	0	5	2	All lobes	
94(40.1%)	34	1	10	2	1	36	10	total	
32 (13.6%)	13	1	1	1	1	9	6	RUL	Right
19 (8.1%)	9	0	1	1	2	6	0	RML	
63 (26.9%)	10	2	8	1	3	31	8	RLL	
15 (6.4%)	4	2	1	0	0	4	4	All lobes	
129 (55.1%)	36	5	11	3	6	50	18	total	
11 (4.8%)	6	0	0	0	0	5	0	bilateral	
234 (100%)	76(32.4%)	6(2.5%)	21(8.9%)	5(2.1%)	7(2.9%)	91(38.9%)	28(11.9%)	total	

LUL: left upper lobe; LLL: left lower lobe; RUL: right upper lobe; RML: right middle lobe; RLL: right lower lobe; N/A: not applicable

**Figure 5:**
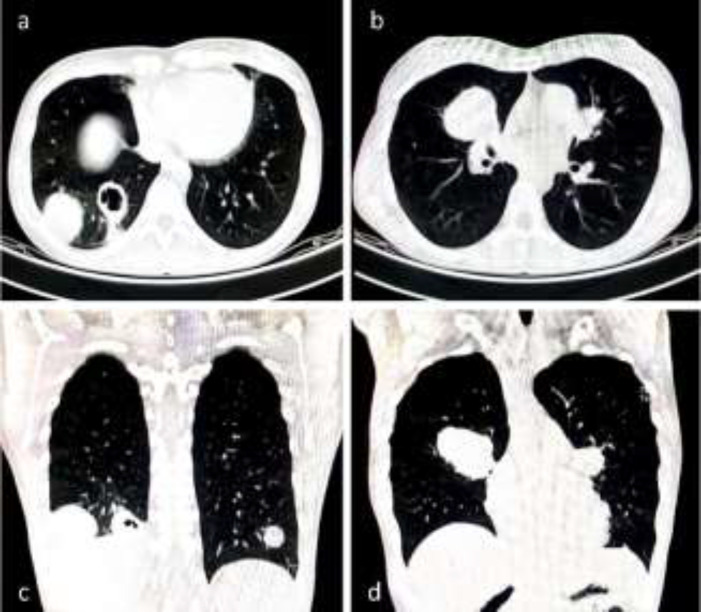
Computed tomography of the chest in axial (a, b) and coronal (c, d) views showed bilateral multiple pulmonary hydatidosis in 28-year-old female patient. The right middle lobe (RML) involvement was more than 70% in this patient. Despite the indication of RML lobectomy, cyst enucleation and broncho-pleural fistula ligation was performed

**Figure 6:**
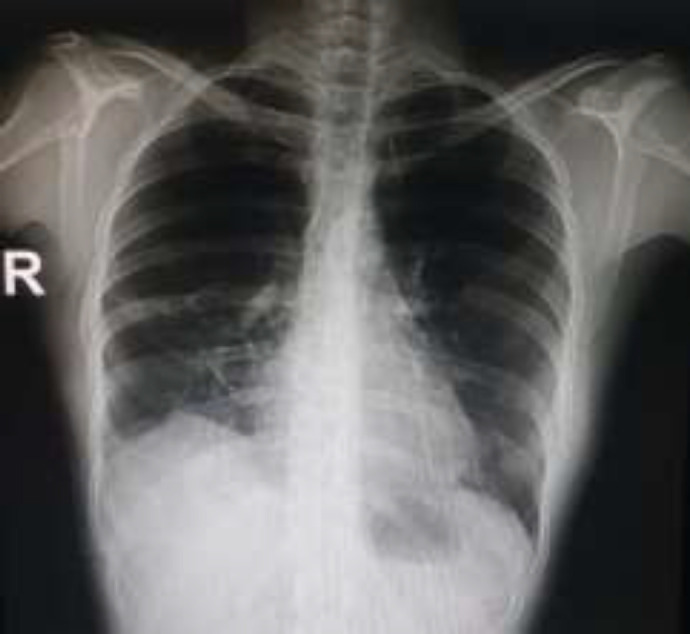
Chest X-ray of the same patient with more than 70% of RML involvement. Note the expansion of the lobe remnant at 1 month after surgery

Surgical interventions included thoracotomy, rigid bronchoscopy, cyst aspiration, cyst unroofing and enucleation, partial wedge resection, broncho-pleural fistula ligation, and lobectomy in some cases. The involvement was estimated to be about 70% in cases of triple segment involvement of the right lower lobe or the left lobes, double segment involvement of the right upper lobe, and more than two-third involvement of the right middle lobe. In this study, 25 (11.9%) patients had these features, with nearly 70% involvement of the lobe. Lobectomy was performed in 5 (20%) cases. In the other 20 (80%) cases, cyst enucleation and broncho-pleural fistula ligation with or without partial wedge resection were performed (figures 5, and 6). Although it was mentioned in the references that hydatid cyst of the lung with more than 50% involvement has indication for lobectomy (9), we conserved most of the lobes with about 70% involvement in our institution, and patients had no postoperative complications (table 2).

**Figure 7:**
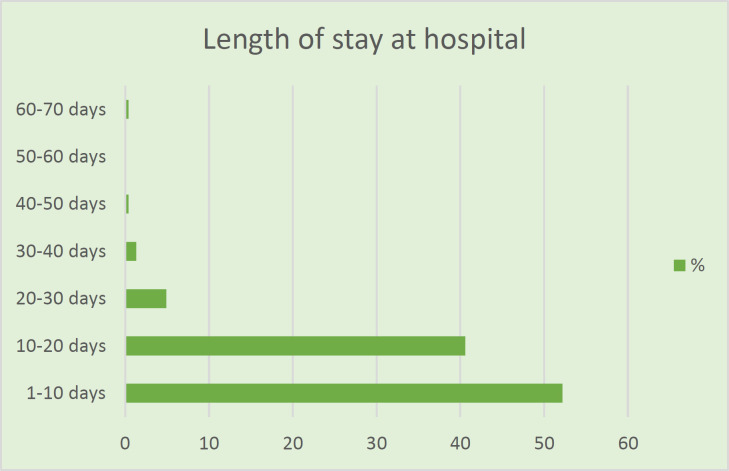
Post-operative length of stay at hospital

Post-operative complications were reported in 14 patients (5.9%). The most common complication was post-operative atelectasis that occurred in 5 patients (35.7%). Pleural adhesions, hemothorax, pneumothorax, dyspnea, fever, and anaphylactic shock were reported in 7.1% of patients. In 3(21.4%) cases, redo thoracotomy was performed due to bleeding, atelectasis, or infection. Based on pathological studies, cysts were at larval stage with walls infiltrated by eosinophils, macrophages, and fibroblasts. Foci of calcification and bronchiolar hyperplasia were noted in some cases. Albendazole was administered for 72 (30%) patients after surgery due to intra-operative rupture or spillage of the cyst fluid. Allergic reaction to albendazole was reported in 1 case. The length of stay at hospital varied from 2 days to 68 days after surgery. 52.2% of patients were discharged within 10 days after surgery. Also, 40.6% of patients were discharged between 10th and 20th days after surgery (figure 7). All patients were discharged in good health condition. No 30-days mortality was reported in this study.

**Table 2:** Surgical interventions for involved lobes

	**Less than 70% involvement** **(n = 209)**	**about 70% involvement** **(n = 25)**
Unroofing and enucleation without partial wedge resection, n (%)	206 (98.6%)	6 (24%)
Unroofing and enucleation with partial wedge resection, n (%)	3 (1.4%)	14 (56%)
Lobectomy, n (%)	0 (0)	5 (20%)

## Discussion

Currently, hydatidosis is one of the most important parasitic diseases in the world. The disease is detected in all five continents with considerable prevalence. The health and economic costs caused by this disease cannot be ignored in most parts of the world, especially in the Middle East, Southern and Central Europe, China, and Africa. In our country, hydatidosis has a high prevalence in endemic areas, especially in the North of Iran (10, 11). Diagnosing hydatidosis is fraught with challenges due to several limitations and barriers. These include the silent course of the disease; avoiding to refer patients either for diagnosis and treatment or to follow-up their condition after treatment; Lack of access to a proper medical system and the need for several diagnostic methods. In the meantime, the records of patients undergoing surgery in hospitals can be somewhat helpful in estimating the extent of the disease and clinical condition, diagnosis, treatment in the community (11, 12).

We observed that pulmonary hydatid cysts were more commonly seen in the fifth decade of patients' life. The prolonged incubation period and latent infection are the main reasons why the disease is more common in the older age group. These people may also be more exposed to the contaminated environment of the host feces (13, 14). In Jordan, the highest rate of infection was in children aged 8-16 years. This difference may be because the infection occurs at younger ages, and the antigen secretion from the cysts at older ages, or it may be because people in the age group of the 40s in that area had higher contact with dogs and the environment around them. However, this study based on radiologic observations, and small cysts did not secrete antigens leading to negative results from serologic studies. This may be due to the early stages of hydatidosis in children, and the cysts are still tiny (15). As mentioned earlier, the lungs are the second most common involving organ in hydatidosis. Pulmonary hydatid cyst is more common in the lower lobes, with the right basal lobe being the most involved (16-18). In the present study, the lower lobes had the most involvement rate (47%) with the RLL being the most involved lobe (26.9%). Bilateral involvement was 4.8% in our study. Chest CT scan and radiography are the usual modalities for pulmonary hydatid cyst detection. The pulmonary hydatid cyst should be seen as a round fluid-filled cyst on CT scan or chest x-ray, unless it is ruptured to the pleural cavity or bronchi. Atelectasis of involved lobe may change the margins of the cyst and create a pneumonia like appearance (16, 19). Floating membranes and loss of spherical shape (slot sign) are usually seen on imaging due to rupture of the cyst (20, 21). Cyst perforation was reported in 39% of cases in the present study. Productive salty sputum, cough, and dyspnea were the most common presenting symptoms in these patients. Infected cyst was also reported in about 3% of patients. Complicated pulmonary hydatid cysts (ruptured or infected) should be differentiated from cavitary lesions, including pyogenic abscess, active cavitary tuberculosis, carcinoma, benign tumors, infarctions, or hemorrhage (22, 23).

There was no pulmonary hydatid cyst recurrence in patients that underwent surgical intervention in our institution. However, six (2.5%) patients had the history of cysts recurrence before surgery. Cyst enucleation was the most common performed procedure in our study (90%). The overall rate of lobectomy was about 2.1%. Usluer *et al*. mentioned that the lobectomy rate was higher in cases of pulmonary hydatid cysts larger than 10 cm in comparison to cyst with diameter smaller than 10 cm (4% vs. 1%, respectively) (24). Lobectomy was not indicated in any of cysts with less than 70% involvement of the lobe in our study. According to previous studies, parenchyma-preserving surgery is preferred to anatomical resections with acceptable rates of post-operative complications. These complications included atelectasis, infection, and air leak in minority of the patients (24-27). We had to perform redo thoracotomy and lobectomy in one patient that underwent parenchyma-preserving surgery. The preserved lobe was not ventilated and became infected in this patient. As the diameter of the cyst increases, more complications are expected. Higher growth rate of hydatid cyst depends on elasticity of the lungs, peripheral locations, and silent course of the disease (26). 

In the present study, albendazole as a scolicidal agent was prescribed in 30% of patients after surgery. It might be concluded that scolicidal administration is unnecessary if the spread of the parasite is avoided during the cyst's evacuation. It should be noted that the intra-operative rupture of a cyst may lead to life-threatening anaphylactic shock, with the mortality rates about 0-23.5% (28-30). Co-administration of albendazole and praziquantel as chemoprophylaxis before surgery is considered more effective than either treatment alone and may reduce the treatment period (31). It seems that the medical treatment of hydatid cyst may be one of the appropriate alternatives to surgery in some patients. On the other hand, due to cases of recurrence and its spread during surgery, the prophylactic use of scolicidal drugs can be useful in preventing recurrence of cysts. Pulmonary hydatidosis is usually referred to solitary cysts with a tendency to lower lobes and peripheral areas involvement. Radiologists should be aware of the atypical findings of a ruptured cyst in different imaging modalities. Most authors believe that the optimal treatment for a pulmonary hydatid cyst is surgery. In references, it is recommended that a hydatid cyst involving 50% of a lobe with abnormal surrounding tissue of the lung has the indication for lobectomy (9). Although we had to do lobectomy in one case because of atelectasis and fever of the patient, we conserved lobes with about 70% involvement in other cases. This allows more alveolar tissue to remain in the lung and affects patients' lifestyle. Cyst enucleation instead of lobectomy seems to be safe and a higher threshold can be set for lobectomy of extensive hydatidosis. 

Our study had some limitations. One of them was the incomplete registration of epidemiological information in some cases, which is one of the disadvantages of studies based on recorded data. Another was the unknown medical or surgical history of patients that referred for pulmonary hydatidosis. 

## Funding:

None declared.

## Conflicts of interest:

The authors declare that they have no conflicts of interests.
